# Occupational Infections Among Workers in Europe: Protocol for a Scoping Review

**DOI:** 10.2196/59606

**Published:** 2025-01-24

**Authors:** Guglielmo Dini, Stefania Curti, Alborz Rahmani, Paolo Durando, Stefano Mattioli

**Affiliations:** 1 Department of Health Sciences University of Genoa Genoa Italy; 2 Occupational Medicine Unit IRCCS Ospedale Policlinico San Martino Genoa Italy; 3 Department of Medical and Surgical Sciences University of Bologna Bologna Italy; 4 Department of Environmental and Prevention Sciences University of Ferrara Ferrara Italy

**Keywords:** infection, work-related, biological hazard, narrative synthesis, Europe, occupational infection, worker, scoping review, infectious, prevention and control, occupational health, epidemiology, burden of disease, phenomenon

## Abstract

**Background:**

Workers may be exposed to different infectious agents, putting them at risk of developing occupational diseases. This can occur in many ways, through deliberate use of speciﬁc microorganisms or through potential exposure from close contact with biological material. Infection prevention and control measures against biohazards can reduce the risk of infection among workers. During the last few decades, an increasing proportion of workers in Europe have been exposed to infectious biological agents in their workplace. Knowledge gaps on this topic in Europe have limited our understanding of the overall phenomenon in occupational settings.

**Objective:**

This study aims to understand the extent and type of evidence on the epidemiology of occupational or work-related infections caused by bacterial, viral, fungal, and parasitical agents in European countries, the factors affecting their occurrence among workers, and the burden of disease among workers due to occupational risk.

**Methods:**

The review will be conducted following the Joanna Briggs Institute methodology for scoping reviews and the PRISMA-ScR (Preferred Reporting Items for Systematic Reviews and Meta-Analyses extension for Scoping Reviews) guidelines. This review will consider studies that include data on the epidemiology of occupational infections, risk factors and determinants, and burden of disease among workers employed in specific occupational sectors in European countries in the period 2010-2023. The search will include MEDLINE, Web of Science, and Scopus databases. Independent reviewers (including GD, SC, AR, PD, and SM) will screen the titles, abstracts, and full texts of the selected studies. Data extraction will be performed using a tool developed by the researchers. The data will be mapped and analyzed according to the type of extracted data.

**Results:**

The literature search through different scientific databases started in April 2024 and is expected to be completed by December 2024. The findings will be extracted using an ad hoc data extraction tool, and relevant results will be presented in narrative and tabular form.

**Conclusions:**

This scoping review aims to provide rigorous evidence to fill the knowledge gap in the epidemiology of occupational or work-related infections in European countries, the factors affecting their occurrence, and the burden of disease in different professional settings. Such findings could improve the understanding of this complex occupational phenomenon in the European context, enabling more accurate and up-to-date surveillance of infections incurred in the workplace.

**International Registered Report Identifier (IRRID):**

PRR1-10.2196/59606

## Introduction

Biological agents include a variety of microorganisms, toxins, and allergens that may harm human health. In particular, microorganisms capable of causing infections, such as bacteria, viruses, and fungi, can be pathogenic or can produce diseases that can be transmitted to individuals through various modes of transmission, determining acute or chronic health conditions [1].

In the occupational setting at the European level, prevention and management of all hazards and risks present in the workplace are required to safeguard occupational health and safety for all workers [2]. From this perspective, infection prevention and control measures implemented in the workplace against biological hazards can reduce the risk of infection among workers.

Indeed, employees in different professional sectors may be exposed to a variety of infectious agents, putting them at risk of disease. This exposure can occur in many ways, either through “deliberate use” of speciﬁc microorganisms (eg, laboratories and biotechnological industries), through “potential exposure” from processes of activities that require close contact with biological material (composting, recycling, and wastewater recycling), through animal contact (agriculture and food processing), or through contact with humans (health care and education). Globally, the overall annual mortality attributable to occupational infections is estimated at approximately 320,000 deaths, 5000 of which occur in the European Union [3]. However, morbidity from work-related infections could be largely underreported in national and international surveillance systems, possibly because of the lack of distinctive characteristics of work-related infectious diseases compared with infections acquired in nonoccupational settings, thereby making it difficult to establish a causal link between work and disease. Nonetheless, work-related infections may result in significant harm to workers’ health, potentially resulting in a high disease burden on the working population. Indeed, experiences in tracking occupational diseases from biological agents are rather different [4], facilitating underestimation of the phenomenon. Indeed, there is often vast heterogeneity between countries in the definition of occupational or work-related diseases or injuries caused by biological agents. The case of SARS-CoV-2 infection among workers is paradigmatic: despite the large literature published on the subject, few countries notify this disease as an occupational accident or injury (eg, China and Italy), while the majority of countries have labeled this event as an occupational disease [5]. In fact, according to the Italian legislation on workers’ compensation, cases of infectious and parasitic diseases are included in the category of accidents at work “because the virulent cause is equated to the violent one,” which is a required characteristic to identify a work-related injury [6,7].

In particular, during the last few decades, increasing proportions of workers in Europe have been reported to be exposed to infectious biological agents in the workplace [8]. This has increased the need to provide more insight into the appropriate study and assessment of biological risks present in European countries, as well as their potential health effects among the workforce, especially in light of the impact of the COVID-19 pandemic on occupational health. Recent reports from the European Agency for Safety and Health at Work have identified several high-risk occupations for occupational infections (eg, animal-related occupations, waste and wastewater management, health care setting, farming, and traveling for work) and have provided recommendations on the implementation of specific preventive and protective measures in these settings. However, due to limited knowledge caused by the lack of up-to-date information on the ecology of pathogens and epidemiology of associated diseases in the European Union, particularly due to data gaps in monitoring systems for occupational diseases in member countries, it has not yet been possible to reach a comprehensive understanding of the overall phenomenon in all occupational settings [9]. Indeed, in many countries, biological agents are often not considered an Occupational Safety and Health priority, resulting in a reactive rather than a proactive approach to recognizing occupational biohazards and managing the risks to the safety and health of workers.

To understand the available knowledge on this topic, a scoping review will be performed to map the existing literature on infectious diseases in the occupational setting, enabling us to gather evidence on both traditional and emerging occupational biological agents and provide useful insights into their determinants and health impact. The scoping review was chosen as the most appropriate type of review as our aim is to achieve breadth rather than depth in our analysis. Therefore, other types of reviews are not deemed methodologically effective [10].

This scoping review is expected to provide the first rigorous analytical and updated synthesis of research data on the epidemiology of injury or disease due to infectious biological hazards, as well as information on the occupational burden. Stratifying infections according to the type of infectious disease and work task involved could add meaningful information and increase our understanding of the risk factors and corresponding determinants. Indeed, filling knowledge gaps and acquiring high-quality evidence concerning the epidemiology of occupational infections in European countries could be used as the scientific basis for developing and implementing effective preventive programs as well as for informing the activities of Occupational Health Services. This would contribute significantly not only to employers, employees, researchers, and occupational health professionals but also to overall public health.

A preliminary search of MEDLINE, the Cochrane Database of Systematic Reviews, and Joanna Briggs Institute (JBI) Evidence Synthesis was conducted, and 2 published systematic reviews on the topic were identified [11,12]. However, these 2 systematic reviews did not specifically report data on the prevalence and incidence of occupational infections, definition of risk factors and determinants, as well as direct and indirect burden of disease (eg, economic impact and days absent from work due to ill health). In addition, the most recent systematic review was limited to non–health care workers and covered the period 2009-2020 [12]. Hence, we decided to perform a new review to gather updated evidence on all occupational categories, including health care settings. Furthermore, we decided to restrict the focus of our review to studies performed in European countries, in consideration of sufficiently comparable economic development and diversification, good standards of health care services, including occupational safety and health requirements provisions, as well as similar microorganism ecology, in order to better grasp the updated scientific evidence and possibly reduce confounders and heterogeneity in the subsequent synthesis.

The objectives of this scoping review are (1) to provide a comprehensive and up-to-date synthesis of all studies concerning occupational or work-related infections in European countries; (2) to identify the factors that impact the occurrence of infections among workers; and (3) to quantify the burden of infection among workers in terms of related disabilities, residual working capability, absence from work, and direct and indirect costs generated.

## Methods

The proposed scoping review will be conducted in accordance with the JBI methodology for scoping reviews [13] and in line with the PRISMA-ScR (Preferred Reporting Items for Systematic Reviews and Meta-Analyses extension for Scoping Reviews) guidelines [14].

### Review Questions

The review questions of our planned study are as follows:

1. What is the incidence or prevalence rate of infections among workers in European countries?
2. What are the determinants of infection among workers in European countries?
3. What is the burden imposed by infections among European workers in terms of related disabilities, residual working capability, absence from work, and direct and indirect costs?

### Eligibility Criteria

Eligibility criteria, defined according to Population, Concept, and Context criteria, are listed in Textbox 1.

**Eligibility criteria defined according to Population, Concept, and Context criteria.**

**Participants**

- Workers employed in specific occupational sectors.

**Concept**

- Epidemiology of occupational infections, associated risk factors and determinants, burden of impact on health in terms of related disabilities, residual working capability, absence from work, and direct or indirect costs.

**Context**

- Studies published since 2010, performed in European countries with a common geographical definition of Europe [15].

**Types of Sources**

- This scoping review will consider all publications that meet the inclusion criteria. This includes, but is not limited to, quantitative, qualitative, and mixed methods studies. Systematic reviews that meet the inclusion criteria will also be considered depending on the research question. If the same data are reported in more than one publication, the primary article or article with the most complete data will be used.
- Analytical observational studies, including prospective and retrospective cohort, case-control, and analytical cross-sectional studies, will be considered for inclusion. Studies not matching the defined Population, Concept, Context criteria, review articles, modeling studies, case series, and individual case reports will be excluded from the review.

### Search Strategy

A 3-step search strategy will be followed, as described in the JBI Manual for Evidence Synthesis [13].

An initial limited search of MEDLINE was undertaken to identify articles on the topic of interest. Text words contained in the titles and abstracts of relevant articles and the index terms used to describe the articles were used to develop a full search strategy for MEDLINE (Multimedia Appendix 1). To improve the specificity of the search strategy for injuries and diseases caused by occupational exposure, we adapted the specific filter developed by Mattioli et al [16] to retrieve potentially pertinent citations. The search strategy, including all identified keywords and index terms, will be adapted for each included database and information source. The databases to be searched will include MEDLINE, Web of Science, and Scopus. The reference lists of all included reports will be screened for additional studies. If the full text of a report cannot be accessed, the authors will be contacted. Studies published in English, Italian, French, and Spanish since 2010 will be included.

The second step will consist of a second search of all included databases using the fields, index, and MeSH terms, and all keywords identified during step 1. The databases to be searched will include MEDLINE, Web of Science, and Scopus. An example of the search strategy, including the index and MeSH terms and keywords and Boolean logic (AND/OR and truncations), is provided in Multimedia Appendix 1.

The third step will consist of screening the reference lists of all the included articles for additional sources. The search strategy will be adapted for each included database and information source.

### Study or Source of Evidence Selection

The source of evidence selection will be completed in the following steps. First, all citations identified through the full search of all included databases will be uploaded into Mendeley Reference Manager 2.91.0, and duplicates removed. Second, titles and abstracts will be screened by 4 independent reviewers to assess the inclusion criteria for the review. Finally, the same 4 independent reviewers will evaluate in detail the full text of the selected articles to verify if inclusion criteria are met. Reasons for exclusion of articles that do not satisfy the inclusion criteria will be reported in the scoping review. Disagreements between the reviewers will be resolved by the fifth reviewer or through discussion to reach a consensus. The complete study inclusion process will be reported in the scoping review and presented in a PRISMA-ScR flow diagram [14].

### Data Extraction

Data will be extracted from the included studies by 4 independent reviewers by means of a data extraction tool developed by the Authors (Multimedia Appendix 2). The extracted data will include details on the participants, concept, context, study methodology, as well as key findings.

### Data Analysis and Presentation

Data analysis and presentation will depend on the data extraction process and potential modification of the data extraction tool; therefore, it will be subject to changes. All changes will be documented in the review. The data will be manually entered into a Microsoft Excel spreadsheet. The data will also include specific details regarding the participants, study methodology, and most relevant findings. If necessary, the draft data extraction tool will be revised during the extraction process, with details on the modifications presented in the scoping review. Relevant results will be presented in tabular form and with a narrative synthesis. Disagreements between the reviewers will be resolved by an additional reviewer or through discussion. All relevant findings will be assessed and discussed by a national collaborative group of researchers from different Italian Universities, Hospitals, and Local Health Authorities.

## Results

The literature search through different scientific databases started in April 2024 and is expected to be completed by December 2024. The review will be submitted for publication in 2025.

## Discussion

The findings of this review will demonstrate an up-to-date epidemiological picture of occupational or work-related infections in European countries, the factors affecting their occurrence, as well as the burden of disease among different professional settings. Recently published data have shown several professional groups at risk of infection, such as the military, livestock farm workers, dairy producers, abattoir workers, and forestry workers, with the majority of these occupational groups being exposed to respiratory pathogens [12]. This review will add to the currently available evidence, by involving studies performed on all working categories, including health care workers. Such findings could improve the understanding of this complex occupational phenomenon in the European context, enabling a more accurate and up-to-date surveillance of infections caused by occupational exposure. This study is strengthened by the comprehensive and rigorous methodological approach adopted in the search strategy. Moreover, the composition of a national collaborative group, gathering expertise from researchers from different Italian Universities, practitioners, and professionals from reference Polyclinic Hospitals, and Local Health Authorities from different Regions of Italy, provides the unique possibility for multifaceted discussions of the findings, offering multiple perspectives from the different actors implicated in Occupational Health. However, the scoping review will be limited by a reduced resolution on the specific types of infections and work categories, as well as by the lack of a quantitative synthesis. To improve both limitations, further targeted systematic reviews and meta-analyses will be necessary. Nonetheless, this scoping review may serve as a foundational resource to inform policymakers with updated evidence, which can be used to improve the definition of occupational diseases and injuries caused by infectious agents in Italy, but also in different European countries. Furthermore, this updated evidence could provide useful practical insights for Occupational Health professionals, as well as guide future research goals by highlighting understudied professional categories and pathogens, with the aim of reducing possible knowledge gaps.

## Appendix

Multimedia Appendix 1 Tentative search strategy.

Multimedia Appendix 2 Data extraction tool.

## Abbreviations

JBI: Joanna Briggs Institute
PRISMA-ScR: Preferred Reporting Items for Systematic Reviews and Meta-Analyses extension for Scoping Reviews

## Data Availability

The datasets generated during and/or analyzed during this study are available from the corresponding author on reasonable request.
