# Supplementary material for: Occupational Infections Among Workers in Europe: Protocol for a Scoping Review
Source: JMIR Res Protoc. 2025 Jan 24;14:e59606. doi: 10.2196/59606 (PMC11806264; doi:10.2196/59606)
Supplement: Multimedia Appendix 1 [file resprot_v14i1e59606_app1.docx]

PCC criteria

| Population (P) | Work* OR Personnel OR Staff OR Occupation* |
| --- | --- |
| Concept (C) | Biohazard OR “Biological Hazard” OR Pathogen OR “Biological Noxa*” OR Parasite OR Infect* OR “Biological risk” OR “Infectious disease[Majr]” OR “Communicable disease[Majr]” |
| Context (C) | Time filter: 01/01/2010-30/11/2023  Geographical filter: Countries present in the common geographical definition of Europe (Albania, Andorra, Austria, Belarus, Belgium, Bosnia and Herzegovina, Bulgaria, Croatia, Cyprus, Czech Republic, Denmark, Estonia, Finland, France, Germany, Greece, Hungary, Iceland, Ireland, Italy, Kazakhstan, Latvia, Liechtenstein, Lithuania, Luxembourg, Malta, Moldova, Monaco, Montenegro, Netherlands, North Macedonia, Norway, Poland, Portugal, Romania, Russia, San Marino, Serbia, Slovakia, Slovenia, Spain, Sweden, Switzerland, Turkey, Ukraine, United Kingdom, Vatican City) |

Tentative search strategy

| **Search** | **Query** |
| --- | --- |
| **1** | infectious agent*[TW] OR biological agent*[TW] OR biologic agent*[TW] OR viruses[MH] OR virus*[TW] OR bacteria[MH] OR bacteri*[TW] OR parasites[MH] OR parasit*[TW] OR helminths[MH] OR helminth*[TW] OR fungi[MH] OR fung*[TW] OR pathogen[TW] OR pathogens[TW] OR protozo*[TW] OR viral*[TW] OR microbiologic*[TW] OR serologic*[TW] |
| **2** | zoonos*[TW] OR mycos*[TW] OR infections[MH] OR infecti*[TW] OR infestation*[TW] OR disease[MH] OR disease*[TW] OR illness*[TW] OR Disease Transmission, Infectious [MH] OR outbreak*[TW] |
| **3** | (occupational diseases[MH] OR occupational exposure[MH] OR occupational medicine[MH] OR occupational risk[TW] OR occupational hazard[TW] OR (industry[MeSH Terms] AND mortality[SH]) OR occupational group*[TW] OR work-related OR occupational air pollutants[MH] OR working environment[TW] OR occupational injuries[MH] OR Accidents, Occupational[MH] OR Needlestick Injuries[MH]) |
| **4** | incidence[MH] OR incidence*[TW] OR prevalence[MH] OR prevalence*[TW] OR occurrence*[TW] OR epidemiology[MH] OR epidemiol*[TW] OR seroepidemiolog*[TW] OR seroprevalence*[TW] OR etiology[TW] OR aetiology[TW] OR burden[TW] OR risk[MH] OR risk*[TW] OR expos*[TW] |
| **5** | #1 AND #2 AND #3 AND #4 |
