# Supplementary material for: Occupational Infections Among Workers in Europe: Protocol for a Scoping Review
Source: JMIR Res Protoc. 2025 Jan 24;14:e59606. doi: 10.2196/59606 (PMC11806264; doi:10.2196/59606)
Supplement: Multimedia Appendix 2 [file resprot_v14i1e59606_app2.docx]

Appendix II. Data extraction tool

| Study reference | Name of authors, year of publication |
| --- | --- |
| Study population | Occupational category, International Standard Classification of Occupation (ISCO) code |
| Country | European country in which the study was performed |
| Study design | Type of study design |
| Gender | Percentage of workers involved stratified by gender |
| Age | Mean age of the worker sample |
| Sample number/attrition rate | Number of workers who took part in the study |
| Professional/experience years | Average years spent in the specific occupation by workers included in the study |
| Working setting | Professional setting where infection occurred |
| Infection prevalence/incidence rate | Prevalence/incidence rates stratified by occupational infection |
| Infection determinants/risk factors | Main determinants or risk factors of acquiring occupational infections by prevalence, risk ratios, odd ratios |
| Infection-related burden | Absenteeism, disabilities, direct/indirect economic costs due to injury/disease, attributable fraction to occupational exposure |
